# SeqCode in the golden age of prokaryotic systematics

**DOI:** 10.1093/ismejo/wrae109

**Published:** 2024-06-19

**Authors:** Diego Javier Jiménez, Alexandre Soares Rosado

**Affiliations:** Biological and Environmental Sciences and Engineering Division (BESE), King Abdullah University of Science and Technology (KAUST), Thuwal 23955-6900, Kingdom of Saudi Arabia; Biological and Environmental Sciences and Engineering Division (BESE), King Abdullah University of Science and Technology (KAUST), Thuwal 23955-6900, Kingdom of Saudi Arabia

**Keywords:** Candidatus names, metagenomics, not-yet-cultivated prokaryotes, prokaryotic nomenclature, prokaryotic taxonomy

## Abstract

The SeqCode is a new code of prokaryotic nomenclature that was developed to validate taxon names using genome sequences as the type material. The present article provides an independent view about the SeqCode, highlighting its history, current status, basic features, pros and cons, and use to date. We also discuss important topics to consider for validation of novel prokaryotic taxon names using genomes as the type material. Owing to significant advances in metagenomics and cultivation methods, hundreds of novel prokaryotic species are expected to be discovered in the coming years. This manuscript aims to stimulate and enrich the debate around the use of the SeqCode in the upcoming golden age of prokaryotic taxon discovery and systematics.

## Brief history of the SeqCode

**Figure 1:**
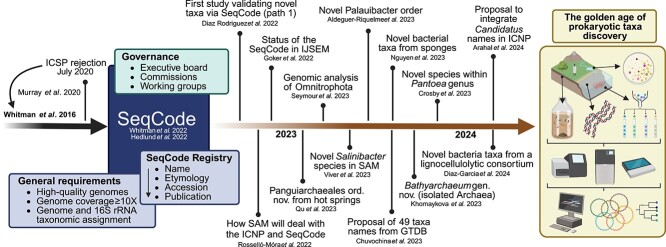
Timeline of the SeqCode. Illustrating the key events and scientific publications surrounding the development of the SeqCode and its use up to march–April 2024. The SeqCode is governed by the SeqCode committee that also oversee the operation of the SeqCode registry. In the SeqCode registry, several general requirements must be followed to create and validate novel taxon names under the SeqCode, such as the use of high-quality genomes, proper formed name and etymology, accession numbers of the assemblies, and effective publication. We predict that the SeqCode will support the next golden age of prokaryotic taxon discovery. In this new era, advances in isolation techniques, top-down enrichment approaches, in situ explorations, cell-sorting methods, long-read DNA sequencing technologies, and new bioinformatic tools will boost the discovery of novel taxa. Abbreviations in the figure: *Systematic and Applied Microbiology* (SAM); *International Journal of Systematic and Evolutionary Microbiology* (IJSEM); International Code of Nomenclature of Prokaryotes (ICNP); Genome Taxonomy Database (GTDB); metagenome-assembled genomes (MAGs). BioRender.com was used to create the figure.

Prokaryotic systematics is considered an important and challenging field in microbiology. For decades, bottlenecks in isolating, cultivating, and analyzing the vast microbial diversity have slowed progress in this field [1]. However, thousands of prokaryotic species have been isolated, named, and maintained in culture collections (e.g. DSMZ-German Collection of Microorganisms and Cell Cultures). A considerable fraction of prokaryotic diversity remains uncultivated, and, probably, many species will be impossible to isolate and/or cultivate in laboratory settings [2, 3]. In 2020, a roadmap for the nomenclature of not-yet-cultivated prokaryotes using DNA sequences as nomenclatural types was proposed [4]. However, the International Committee on Systematics of Prokaryotes (ICSP), which oversees the International Code of Nomenclature of Prokaryotes (ICNP), rejected the original proposal to use DNA sequences as types [5, 6]. At present, only axenic and viable strains deposited in two international culture collections are recognized as nomenclatural type or type material in the ICNP (Rule 30) [7]. In response to the ICSP decision, and after two well-attended online workshops (https://www.isme-microbes.org/seqcode-workshops), the SeqCode was developed in 2022 as a new system for prokaryotic nomenclature that uses genomes (e.g. metagenome-assembled genomes—MAGs) as nomenclatural types [8, 9] (Fig. 1).

## Current state of the SeqCode

The SeqCode enables the creation and validation of permanent names for pure cultures and/or not-yet-cultivated prokaryotes, including taxa denoted as *Candidatus* (a status under ICNP for putative novel taxa) [9]. In addition, the SeqCode also protects the legacy of *Candidatus* names when validated under the SeqCode (i.e. *Candidatus* names are not valid and do not have priority in ICNP and therefore can effectively be overwritten by valid names) [10]. A key feature of the SeqCode is that it recognizes names validly published under the ICNP, and names that are validly published under the SeqCode compete for priority with names validly published under the ICNP after January 2022 [9, 11]. The SeqCode operates via the SeqCode Registry (https://seqco.de/), an online platform to centralize, validate, and compile information about taxon names, etymology, sequences, and publications (Fig. 1).

Although the SeqCode is not recognized by the *International Journal of Systematic and Evolutionary Microbiology* (IJSEM) [12] and some concerns have been raised since its creation (e.g. lessening the importance of isolating pure cultures, thereby indirectly restricting the bioresource use of prokaryotes) [13], the SeqCode has started to be widely embraced by microbial ecologists, taxonomists, journals, and established resources. For example, the International Society for Microbial Ecology (ISME) supports and hosts the SeqCode initiative (https://www.isme-microbes.org/seqcode-initiative), and the Genomes Online Database (GOLD) has stated its plans to incorporate the nomenclature regulated by the SeqCode [14]. In addition, some subgroups of the ICSP have recognized and supported the use of the SeqCode; for instance, the Subcommittees of Chlamydiae, Rhizobia, and Agrobacteria [15, 16]. However, these subcommittees are not independent of the ICSP and can only consider taxon names as valid if they have been validly published under ICNP.

At present, the SeqCode is governed by a committee (https://seqco.de/committee) composed of different commissions, working groups, and an Executive Board that ensure the suitable use of this nomenclature system. In addition, these bodies promote the adoption and community engagement of the SeqCode via online sign-up forms (https://seqco.de/join), social media (e.g. Twitter/X; @seq_code), Slack, scientific publications, meetings, and outreach activities [17]. Recently, a SeqCode prize (https://seqco.de/prize) was created to recognize significant advances in prokaryotic systematics using the SeqCode. Social media promotion (e.g. Twitter/X) is an effective way to engage the community. This can be utilized more by the committee to increase the adoption of the SeqCode. Up to April 2024, ~100 articles (in Google Scholar) had cited the SeqCode paper [9], reflecting the impact and relevance of this alternative code of prokaryotic nomenclature.

## Pro et contra of the SeqCode

Several arguments have been depicted against the use of genomes as nomenclatural types, for instance, genomes are not error free, they can evolve, and they do not contain all the information about the physiology, morphology, and ecology of the entire prokaryotic organism [18, 19]. This is supported by the fact that many prokaryotic genes are uncharacterized and do not have a functional annotation. However, an in-depth and accurate analysis of genomes can potentially inform some functional traits and phenotypic signatures of prokaryotic taxa. Currently, specific bioinformatic tools can provide information about functional traits based on genome sequences and (meta)transcriptomics data (e.g. microTrait and TbasCO) [20, 21]. These approaches can deepen our understanding of the ecological roles, metabolic processes, and adaptive strategies of prokaryotes in diverse ecosystems, generating valuable information for targeted cultivation. Here, the question becomes: to what extent is a comprehensive functional description needed to validate prokaryotic taxon names using genomes? In accordance with other scientists [11, 22], we concur that prokaryotic genomes are extremely useful for species circumscriptions, for unambiguously identifying a taxon, and for suitable nomenclatural types in systematics.

The discussions about unifying (or not) the ICNP and the SeqCode are in progress. However, these two nomenclature codes are still incompatible. Recently, a new proposal to integrate *Candidatus* names in the ICNP was published, which included a very critical point of view about the implementation and use of the SeqCode [19]. In this proposal, the authors state that the SeqCode will generate instability, ambiguity, and confusion in prokaryotic systematics. In addition, they questioned the SeqCode’s representativeness [19]. In contrast, supporters of the SeqCode have criticized Rule 30 in the ICNP [7]. Most of them are brought about due to the known issues in the isolation and cultivation of many prokaryotic taxa, the high financial cost associated with deposition and maintenance of pure cultures, and the barriers to fulfill this rule in countries where legislation does not support the exportation of biological material [8, 9, 11, 22]. In this regard, we predict that many researchers who have trouble exporting axenic cultures will decide to use the SeqCode for the validation of taxon names. As an example, a novel species of the genus *Fictibacillus* isolated from Brazilian Amazon dark soils [23] was validated using the SeqCode instead of ICNP (https://seqco.de/r:yknhgrq2). In the same way, seven names of novel *Mesorhizobium* species, isolated from root nodules of *Vachellia karroo* in South Africa, were validated via the SeqCode Registry [24]. Overall, these different points of view—pros and cons—reflect the ongoing discussion among microbiologists concerning this topic.

## Use of the SeqCode to date

Currently, two paths can be followed to validate novel taxon names via the SeqCode Registry: (i) registration of unpublished taxon names and independent peer review and (ii) registration of existing taxon names (e.g. *Candidatus*) from published studies. A third path will be implemented with partner journals, including integration of the SeqCode registration and peer review process [9, 11]. These three paths are only methodological and all valid names via SeqCode will have the same significance.

The earliest adopters of the SeqCode (via path 1) reported two novel bacterial taxa (*Pristimantibacillus lignocellulolyticus* gen. nov., sp. nov., and *Ochrobactrum gambitense* sp. nov.) using single-contig MAGs obtained from the metagenome of a minimal lignocellulolytic microbial consortium [25] (Fig. 1). This reduced community was derived from a complex bacterial consortium (named T6) via the dilution-to-stimulation/extinction method. The Andean soil-derived T6 consortium was also subjected to further studies leading to the descriptions of other novel bacterial taxa, including *Andeanibacterium colombiense* gen. nov., sp. nov., and 14 novel species, which were validated using the SeqCode Registry [26] (Fig. 1; Table 1). These two studies [25, 26] coincided with the startup of the SeqCode and exemplify the SeqCode Registry’s ability to validate the names of prokaryotic taxa in an easy, efficient, and timely way.

**Table 1:** List of example studies that have proposed and validly published novel taxon names under the SeqCode-after its publication in 2022 [9], including isolation sources, journals, number of taxon names, and registry accession links.

**Year**	**Month**	**Isolation sources**	**Number of novel taxon names**	**SeqCode registry accession**	**Journal**	**Reference**
2022	September	Forest soil	3 new names including *Pristimantibacillus* gen. nov	seqco.de/r:xwx6hrsf	ISME Communications	[25]
2023	March	Hot springs	4 new names including *Panguiarchaeales* ord. nov.	seqco.de/r:d51mzlo9	Cell Reports	[27]
		Marine sediments and water	*Blastopirellula sediminis* sp. nov. *	seqco.de/r:du78m-se	Antonie van Leeuwenhoek	[28]
		Various sources	*Omnitrophus fodinae* sp. nov. and *Omnitrophus* gen. nov.	seqco.de/r:d841ck02	Nature Microbiology	[29]
	May	Saline Brines	3 names including *Salinibacter pepae* sp. nov.	seqco.de/r:b5vsvzg3	SAM Journal	[30]
	June	Marine sediments, sponges, and saline soils	25 new names including *Palauibacterales* ord. nov.	seqco.de/r:0hkazsoc	mSystems	[31]
	July	Various sources	42 new names including *Binataceae* fam. nov.	seqco.de/r:3yxqlvua	FEMS Microbiology Letters	[32]
		Various sources	6 new names including *Hadarchaeaceae* fam. nov.	seqco.de/r:7ewkque5		
		Beetles gut	3 new names including *Bostrichicola* gen. nov.	seqco.de/r:vppriic7	The ISME Journal	[33]
		Sponges	16 new names including *Spongiisociales* ord. nov.	seqco.de/r:v1sky3wb	SAM Journal	[34]
	August	Human feces	*Intestinicoccus colisanans* gen. nov. sp. nov. *	seqco.de/r:tpd6ryk0	BMC Research Notes	[35]
		Sediments	*Bathyarchaeum tardum* gen. nov., sp. nov. *	seqco.de/r:0qcnd580	Frontiers in Microbiology	[36]
	September	Hot springs	4 new names including *Pelearchaeum* gen. nov.	seqco.de/r:-286wyu6	Frontiers in Microbiology	[37]
		Mouse gut	*Taurinivorans muris* gen. nov. sp. nov. *	seqco.de/r:pzblpla3	Nature Communications	[38]
	October	Soda lake and a terrestrial mud volcano	3 names including *Methanocrinis harundinaceus* gen. nov. sp. nov.	seqco.de/r:gyelqp06	Frontiers in Microbiology	[39]
	November	Various sources	16 new names including *Pantoea alvi* sp. nov.	seqco.de/r:xfaladud	Frontiers in Microbiology	[40]
		Various sources	6 new names including *Sacchlamyda saccharinae* sp. nov.	seqco.de/r:bsk8pkm4	SAM Journal	[41]
	December	Rhizosphere	*Nocardia canadensis* sp. nov. *	seqco.de/r:4msicqid	Microorganisms	[42]
	January	Sediments	3 new names including *Electrothrix* gen. nov.	seqcode.r:lmyvwfoa	SAM Journal	[43]
2024		Forest soil	16 names including *Andeanibacterium* gen. nov	seqco.de/r:-kiq_w89	SAM Journal	[26]
		Corals	*Sororendozoicomonas aggregata* sp. nov. *	seqco.de/r:oe5jqwk0	The ISME Journal	[44]
	February	Human gut	18 new names including *Enterococcus mansoni* sp. nov.	seqco.de.r:csh66vwa	PNAS	[45]
	March	Human gut	3 new names including *Ruminococcus primaciens* sp. nov.	seqco.de.r:636yhucc	Science	[46]
	April	Groundwater	8 new names including *Costitxia debesea* sp. nov.	seqco.de.r:4u3eoyk5	SAM Journal	[47]

^*^Cultivable microbial species; SAM, *Systematic and Applied Microbiology*; ISME, *International Society for Microbial Ecology Journal*.

In March 2023, the ubiquitous phylum *Omnitrophota* (one of the oldest and smallest types of bacteria) was highlighted in the first large-scale genomic analysis to utilize the SeqCode [29] (Fig. 1). In this study, 36 novel species and 4 classes (“*Velamenicoccia*,” “*Omnitrophia*,” “*Gorgyraia*,” and “*Aquiviventia*”) were reported and named [29]. All these novel taxonomic names will be validated once the *Omnitrophus* genus name is granted an exception (see below) for validation by the Executive Board of the SeqCode (https://seqco.de/r:d841ck02). The SeqCode has also been used to validate the names of archaeal taxa. For example, a novel order (*Panguiarchaeales*) was proposed based on MAGs derived from hot springs [27]. In addition, the names of several novel archaeal genera (e.g. *Wolframiiraptor*, *Pelearchaeum*, *Methanocrinis*, *Calditenuis*, and *Bathyarchaeum*) have been validated using the SeqCode Registry [36, 37, 39, 48] (Table 1). In July 2023, 49 names for GTDB-defined higher prokaryotic taxa (based on 23 genomes designated as types) were proposed and validated through the SeqCode Registry [32] (Table 1). In addition, a comprehensive analysis of *Pantoea* genomes [40] allowed the identification of novel species whose names were validated under the SeqCode (Fig. 1; Table 1). Up to March 2024 (when this perspective was developed), 221 prokaryotic taxon names have been validated via the SeqCode Registry, including nine new orders (e.g. *Palauibacterales* from marine sediments) [31], 16 families (e.g. *Spongiisociaceae* from sponges) [34], 60 genera (e.g. *Sororendozoicomonas* from corals) [44], and 122 species (e.g. *Salinibacter pampae* from saline brines) [30] (Table 1). Most of these names were validated through path 1, and others were previously designated as *Candidatus* (e.g. *Macondimonas* from oil-contaminated sediments and *Elulimicrobium* from freshwater) [49, 50]. Because the SeqCode allows validation of the names of cultivated and/or not-yet-cultivated taxa, we predict that these numbers will increase exponentially in the coming years. To exemplify this, from March 2024 to April 2024, around 123 prokaryotic taxa names were validated via SeqCode Registry. A complete list of validly published names can be found on the SeqCode Registry platform.

In 2023, studies validating novel taxon names using the SeqCode were published mostly in journals, such as *Systematic and Applied Microbiology* (SAM), ISMEJ, and *Frontiers in Microbiology* (Table 1). Some studies have mentioned the use of the SeqCode (e.g. [51–54]), but registry links were not found, and their reported taxon names are probably under validation process. From our point of view, the inclusion of these links must be required in the manuscripts that use the SeqCode. Accordingly, journals that choose to adopt or incorporate the SeqCode can append this requirement in the author guidelines. The National Center for Biotechnology Information (NCBI) is now providing links to the available SeqCode Registry lists on its taxonomy pages (e.g. https://www.ncbi.nlm.nih.gov/Taxonomy/Browser/wwwtax.cgi?id=3056650&mode=info). Moreover, the online platform of the SeqCode Registry is easy to navigate and user-friendly. However, it would be very helpful if the name list and/or register list included (as an additional field) the path number used for taxon names validation and source of those species (e.g. axenic cultures, MAGs, or both). Although the path number does not affect the status of the names, this would facilitate upcoming metadata analyses and enable efficient searching of taxon names by linking them to paths, sources, and studies.

## Genomes features

In brief, to validate species names via the SeqCode Registry, several requirements must be followed, e.g. use of high-quality genomes (i.e. >90% complete and <5% contamination), genome read coverage ≥10X for isolates, availability of the assemblies in INSDC (The International Nucleotide Sequence Database Collaboration) databases, evidence of species uniqueness, and correctly formed name and etymology [9, 11] (Fig. 1). Although lower-quality genomes (e.g. higher-contaminated) could be useful as nomenclatural types [55], keep these high standards ensuring unambiguous taxa identification, a proper functional annotation of the nomenclatural genomes, and makes the SeqCode a more robust system.

The completeness of a genome is commonly determined by detecting the presence and copy number of universal or taxon-specific sets of marker genes, e.g. as implemented in the ubiquitously used CheckM software [56] or MiGA [57]. However, novel prokaryotic taxa with reduced genomes could lack some of these marker genes, affecting their completeness percentages. Fortunately, some recent machine learning methods (i.e. CheckM2) could overcome this limitation [58]. In this regard, SeqCode curators would consider low genome completeness values in particular cases. In June 2023, a public discussion (https://github.com/seq-code/seqcode/discussions/2) was conducted on the use of an incomplete genome (~70% complete) to validate the name *Omnitrophus fodinae* under the SeqCode. After several months of discussion between the reconciliation commission, the executive board, and the scientific community, this exception was granted to retain the nomenclature of the entire phylum and the name *Omnitrophota* (https://seqco.de/r:6keafw8d). These types of cases must be treated with caution, and proper public discussions are advocated to generate useful arguments for the final decision by the reconciliation commission. Moreover, the SeqCode developers recommend high genome integrity (i.e. contig no. < 100; N50 > 25 kilobases (kb); largest contig > 100 kb) for nomenclatural types. With recent advances in long-read DNA sequencing technologies (e.g. PacBio and Oxford Nanopore), many single-contig MAGs will be used for new taxon descriptions, as exemplified by a recent study [26]. The SeqCode developers also recommend avoiding descriptions of novel taxa based on single high-quality MAGs derived from a single sample [9]. This may ensure the nonchimeric origin of the genomes. However, in some cases, obtaining more than one biological replicate poses a challenge, for instance when acquiring fecal samples from an individual or a particular specimen.

In January 2023, the editors of the journal SAM described the minimum requirements for new taxon descriptions under the SeqCode and ICNP [59]. The SeqCode recommends an agreement between the genome and 16S rRNA gene taxonomic assignments [9]. This recommendation is a mandatory rule in the journal SAM. However, 16S rRNA gene sequences are often missing from fragmented MAGs because of their conserved nucleotide composition and multiple copies [60]. This issue must be considered by the SeqCode committee and other prospective partner journals that will incorporate the SeqCode (i.e. via path 3). Additionally, we noticed some mismatches between the quality criteria suggested by the SeqCode developers and the SAM journal. For instance, MAGs read coverage ≥10X and the presence of the 16S rRNA gene (at least 75%) are required in the SAM journal to publish novel taxon names under the SeqCode [59]. These features are suggested by the SeqCode developers, but they are not mandatory [9]. At this point, some questions remain unanswered. For example: (i) Which additional journals will adopt the SeqCode? (ii) Will the requirements suggested by the SeqCode developers be the same as those adopted for partner journals? (iii) Will it be necessary to create a specialized journal for this purpose?

## Species circumscription

To validate the name of novel prokaryotic taxa under the SeqCode, it is essential to provide clear evidence of the species’ uniqueness, including its taxonomic rank and position [9]. There still is no clear prokaryotic species concept that is agreed upon, yet major progress has been made through systematics and genome analyses [61]. In this regard, it is widely accepted that genomes sharing average nucleotide identity (ANI) values >95% belong to the same species, and those with average amino acid identity (AAI) values <65% can be assigned to novel genera or even higher taxonomic ranks [62]. Furthermore, digital DNA–DNA hybridization (dDDH) values <70% can support these thresholds [60, 63]. From our experience, MiGA [57] and TYGS [64] are user-friendly online platforms that can provide genomic relatedness indexes (e.g. ANI, AAI, and dDDH) useful for species circumscription. Additionally, to confirm the uniqueness of prokaryotic species (as well as for higher taxa), a monophyletic origin is required. In this scenario, to accurately determine the taxonomic placement of putative novel MAGs, the use of phylogenetic reconstructions based on concatenated single-copy marker genes or core proteins (e.g. those generated using GTDB-Tk v2 [65] or PhyloPhlAn 3.0 [66]), is commonly performed. Recently, Riesco and Trujillo [63], have discussed thresholds to delimitate new species and genera based on genomic relatedness indexes and phylogenomic trees, updating minimal standards for the use of genomes in the taxonomy of prokaryotes.

## Name formation

As mentioned above, a name must be formed according to the rules with an appropriate etymology to be considered for validation. In the SeqCode, the use of Latin is mandatory and can facilitate its compatibility with ICNP [9, 11]. This requirement can be a drawback for many microbiologists lacking proper Latin skills. Fortunately, a function of the SeqCode Registry and nomenclature group is to provide advice to those needing assistance in forming Latin names for novel taxa. The SeqCode curators are also authorized to correct any typographical or orthographical errors during validation, similar to the IJSEM practice [10]. This procedure can ensure an accurate name and etymology, thereby avoiding mistakes and retractions in taxon names. Recently, the SeqCode developers have listed simple rules for creating Latin prokaryotic taxon names [11]. However, if hundreds or thousands of new prokaryotic names are validated under the SeqCode, it is unclear how curators will keep pace. Automation inside the SeqCode Registry will be very useful to tackle this issue. In this regard, the use of an automatic generator of taxon names (called GAN) [67] and other tools (e.g. protologger) [68] could facilitate the description of novel taxa, providing helpful information (e.g. names, etymology, and ecological features) to write taxon protologues [11, 69]. Moreover, a crucial consideration is that if a *Candidatus* name for the taxon already exists, an attempt should be made to keep the name as much as possible, even if it is not required under the rules of the SeqCode. This will improve communication, leading to a more stable nomenclature.

## New era for prokaryotic taxon discovery

A comprehensive catalog of MAGs from different microbiomes revealed that thousands of microbial species are waiting to be discovered [70]. In this context, we predict that recent advances in long-read DNA sequencing technologies, cultivation methods, computational tools, and the SeqCode initiative will kick-start the upcoming golden era of microbial taxon discovery and prokaryotic systematics. This will open new possibilities for understanding microbial dark matter, expanding our knowledge of prokaryotic diversity and function (Fig. 1). In this regard, some approaches will boost the discovery of novel prokaryotic taxa from environmental microbiomes. For example: (i) *in situ* explorations (using deployable DNA extraction and sequencing technologies) can provide a real-time overview of microbial communities, avoiding biases associated with sample storage and transportation [71]; (ii) *ex situ* perturbation experiments (e.g. microcosms) can reshape the original microbial communities, increasing the chance to detect novel species; (iii) the design of top-down selective approaches (e.g. enrichment cultures) can generate microbial systems with specific types of prokaryotes that would be difficult to detect directly in nature [26, 72]; (iv) the use of single-cell sequencing and mini/midi metagenomics can enable the recovery of unseen microbial populations [73, 74]; (v) the use of innovative cultivation techniques (e.g. *in situ* devices) [75], genome-scale metabolic models and culturomics [76] can improve the isolation of yet-uncultivated species; and (vi) hybrid assemblies between long-read and short-read DNA sequencing data can improve the recovery of high-quality MAGs, allowing analysis at the intraspecific or strain level [77, 78]. In this new era, innovative bioinformatics tools will facilitate the analysis of large-scale genomic datasets, allowing microbiologists to infer phylogenetic relationships and taxonomic placements with unprecedented accuracy. Overall, the use of genome sequences as type material to create and validate taxon names will shape and revolutionize future research in prokaryotic systematics. In this sense, the SeqCode will be essential in upcoming microbiome studies, and its broad acceptance can improve the communication between microbiologists.

## Future of the SeqCode in prokaryotic systematics

For the benefit of the scientific community, we hope that the SeqCode and ICNP can be merged to avoid confusion in nomenclature and instability. In this regard, the recent proposal to integrate *Candidatus* names into the ICNP [19] can be a starting point. In fact, this proposal is similar to what the steering committee of the SeqCode wanted to achieve initially. The SeqCode is partially compatible with the ICNP in many aspects [11]. This compatibility can be very helpful for creating unified taxonomies of cultivated and not-yet-cultivated prokaryotes. Given that genome sequences are available for most type strains, the merging of SeqCode and INCP could be effortless. From our opinion, the proposal to include *Candidatus* names in the INCP is still confusing and does not address the central issue of providing an equal nomenclatural standing for cultivated and not-yet-cultivated prokaryotic taxa. All the significant aims of this proposal [19] can be achieved by recognizing the SeqCode in the ICNP. In the case that both codes cannot merge, effective communication is necessary to ensure non-ambiguity in taxon names validated by genomes and those validated by pure cultures. The simplest way to ensure harmony between the codes is for ICNP to recognize validly published SeqCode names, as SeqCode already does for ICNP names. In this regard, it is demonstrated that both codes can work in parallel [30]. Finally, it remains unclear how the SeqCode will be funded, self-sustained or self-supported in the coming years, and if the users or institutions will need to pay fees to use it. In any case, only time will tell what will be the future of the prokaryotic systematics and the SeqCode in this predicted golden age of taxa discovery.

## Data Availability

No datasets were generated or analyzed during the current study.
